# Christmas Thoughts at the National Gallery

**Published:** 1892-12-24

**Authors:** 


### Christmas Thoughts at the National Gallery.

It is interesting to notice the diversity in conception of the early painters with regard to well-known Scripture scenes. In modern days, when the principal attempt is to render the scene as real as possible, and attain something like a faithful representation of the costume, buildings, and customs of ancient Judea, this diversity is somewhat less apparent. And yet, if the early painters were not hampered by nice notions of archæology they were often guided by convention, and followed in main features the scheme adopted by some celebrated predecessor. The scene of the Last Supper, for instance, was invariably placed by the Italian painters in the refectory of a monastery until Tintoretto daringly broke the tradition.

The National Gallery contains five principal pictures of the Nativity, or, perhaps, it would be more exact to say of the Adoration of the Shepherds. "The Adoration of the Magi" is a distinct group, and of this, as opening up wider possiblilities of grouping and effect, there are many more examples. At this season it may not be amiss to spend an hour in the Gallery with the special object of gaining some insight into the methods and conceptions of these five painters with respect to the birth of Christ.

Earliest in date by at least a hundred years is the curious composition of Orcagna (fourth room). The centre of the picture is occupied by a kind of lean-to shed, the roof strangely out of perspective. Within is a strong wooden cradle partly covered by a scarlet drapery, which is the leading point of colour in the whole. The Babe is looking placidly up at the Virgin, who kneels in adoration at the right. On the other side of the cradle an ox and an ass are sitting up on their haunches like dogs, with tails curled symmetrically over their bodies; they are gazing at the Infant with funny, snug faces of self-satisfaction; Joseph adores at the foot, and angels hover above. The shepherds are represented by two cowled monks kneeling with awe; and above, as a kind of supplement, the angels are descending to two shepherds, who are watching some wooden-looking sheep; another animal which may be meant for a wolf or a fox is introduced among the sheep.

Now some or all of the main features of the above we find reproduced in each of the three other Italian representatives of the Nativity which the Gallery affords—the shed, the ox and ass, the kneeling attitude of the Virgin, the attendant angels, and the introduction in a kind of side scene of the annunciation to the shepherds. Yet though retaining something as has been said of the same general design, the work of Pietro della Francesca (908), which comes next in order of time to that of Orcagna, presents in almost every respect a striking contrast. The enormous advance in the art of painting which the lapse of a hundred years had effected is at once apparent. Needless to say, the shed is in correct perspective, for that is Francesca's strong point. The Babe lies naked on blue drapery in the centre. The Virgin in blue and crimson adores Him. Five angels in blue and white stand singing in a formal row behind to the accompaniment of their guitars, while the shepherds, this time real shepherds, are grouped at the side. The oxen are there among the angels. Notice the solitary swallow perched on the roof of the shed. There is another bird on the ground amid a formal garden of clipped hedges and flower beds without colour. On the right is a glimpse of architecture, probably that of the town for which the picture was painted. The tone of the whole is intensely cold and pure.

Again the contrast is extreme when we turn to the glowing symbolism of Sandro Botticelli (third room). Here the strong point is the angels. The Babe in His cradle and the adoring Virgin present no new features, nor do the shepherds grouped on either hand with the inevitable ox and ass looking on at the strange occupants of their stalls. But angels are everywhere. And such angels, with flowing hair and sweet pure faces and graceful majesty of motion, as Botticelli alone has painted. High above in the luminous sky they are interlaced in rhythmic dance; close over the roof of the shed three more are floating. They mingle with the shepherds on either side below the stiff line of trees which marks out the landscape, and again at the bottom of the picture they are seen embracing three figures in symbolic reference to the descent of "Peace on Earth, Goodwill towards Men." Two vicious little devils are gnashing their teeth below in futile wrath among the rocks, seeming to typify that the powers of darkness though shaken are not yet finally subdued. Of all the five this picture conveys the most vivid impression of the Divinity of the scene. The mystery of the Incarnation is here; the sense of the revelation of unseen things which the other painters have all failed more or less to render.

We need not linger long over the elaborate composition of Luca Signorelli (1133), beautiful though it is in many respects. He has crowded into it an almost endless variety—descending angels, a glimpse of the sea, an ancient castle, the shepherds awaking to the angels' hymn, a view of the chapel for which the picture was painted, with crowds flocking into it, then at length the central group. The Babe is on a cushion; the flesh tints are coarse and hard, and the figure is unattractive; a brilliant foreground of flowers surround Him, and the usual figures complete the scene.

And now we leave the Italian painters, and turn to Velasquez. Again an interval of more than a hundred years has elapsed. All is changed. We are in a real stable, so dark that it is difficult to distinguish the figures, even with the light from the open doorway, through which a glimpse of a far distant landscape and flying angel is visible. Here is no conventional Virgin worshiping her babe, but a blooming Spanish maiden holding back the drapery with all a mother's pride in her lovely boy. He has soft, fair hair, and lies in a pretty baby attitude of repose in the coarse manger among the straw. His white robe is bound with lines of red ribbon, which also encircles His head. The shepherds are crowding round, but as the eye becomes accustomed to the subdued light, there is a little shock of surprise at the discovery that some are women. Why not? Still it is peculiar. That old woman with the strongly marked face and high cap is a typical Spanish peasant. A younger woman is blocking the doorway, bearing on her head a high basket from which two strange looking birds, perhaps doves, are peeping. The lad in front of her is bearing a basket of loaves and an offering of dead game. On a sheaf of corn at the foot of the cradle are two sheep, one dead, with a cord attached to its feet. The live beasts of the stall are no longer represented. The early Italian painters loved to show the brute creation rejoicing in the blessings showered upon man, and they freely painted cats, dogs, and other live creatures into the most sacred subjects. In this show of slaughtered animals there is something entirely out of accord with the spirit of the Babe of Bethlehem, and increased study of the picture confirms the first impression that what has been gained in art has been lost in religious feeling. There is, in fact, nothing in the picture, if we except the far distant angel, to suggest its sacred character. We must retrace our steps, and take one more glance at the Botticelli to help us to realise the true significance of Christmas morning.

In the midst of arduous work, especially that dealing with the degradation and disease of the human form, there are few more ennobling exercises than to turn the mind resolutely from the present, and rest among those higher forms of beauty conceived by the painters of a less harassed age. They do not demand a painful critical attitude. They will not preach us a sermon, or even, perhaps, throw much light on the story they attempt to tell. But as we pause under the great colonnade, before returning to the world of omnibuses and mud, of absorbing duties, and the never-ending struggle with time, we are conscious of having been raised for the moment high above it all to a serener atmosphere, for our good.

